# Fluid loading in abdominal surgery - saline versus hydroxyethyl starch (FLASH Trial): study protocol for a randomized controlled trial

**DOI:** 10.1186/s13063-015-1085-3

**Published:** 2015-12-21

**Authors:** Emmanuel Futier, Matthieu Biais, Thomas Godet, Lise Bernard, Christine Rolhion, Justine Bourdier, Dominique Morand, Bruno Pereira, Samir Jaber, for the FLASH trial management committee

**Affiliations:** CHU de Clermont-Ferrand, Département Anesthésie et Réanimation, Pôle Médecine Périopératoire (MPO), Hôpital Estaing, 1 place Lucie Aubrac, 63001 Clermont-Ferrand cedex 1, France; CHU de Bordeaux, Service Anesthésie et Réanimation III, Unité de Neuro-Réanimation, Hôpital Pellegrin, Tripode, Bordeaux, F-33076 France; CHU de Clermont-Ferrand, Pôle Pharmacie, et EA 467 C-BIOSENSS, BP 10448, Clermont-Ferrand, F-63003 France; CHU de Clermont-Ferrand, Direction de la Recherche Clinique (DRCI), Clermont-Ferrand, F-63003 France; CHU de Clermont-Ferrand, Biostatistics unit, Direction de la Recherche Clinique (DRCI), Clermont-Ferrand, F-63003 France; CHU de Montpellier, Département Anesthésie et Réanimation B (DAR B), Hôpital Saint-Eloi, et INSERM U-1046, Montpellier, F-34295 France

**Keywords:** Fluid therapy, isotonic saline, hydroxyethyl starch, goal-directed fluid therapy, surgical patients, abdominal surgery, postoperative morbidity

## Abstract

**Background:**

Inappropriate fluid therapy during surgery is associated with significant morbidity and mortality. Few studies have examined the effects of particular types of fluids (crystalloid or colloid solutions) in surgical patients, especially with the goal of hemodynamic optimization. Isotonic saline is the most commonly used fluid worldwide but may be associated with potential nephrotoxicity. Hydroxyethyl starch (HES) solutions are widely used in surgical patients as a component of goal-directed fluid optimization strategies, but several large multicenter studies have suggested increased rates of acute kidney injury and adverse events with the use of HES in ICU patients. Despite what may be inferred from physiological studies, the benefit and harm of 0.9 % saline and HES during hemodynamic therapy have not been clearly established in surgical patients.

**Methods/Design:**

The FLASH trial is an investigator-initiated, prospective, multicenter, randomized, double-blinded, two-arm trial, randomizing 826 patients with moderate-to-high risk of postoperative complications to receive 6 % HES 130/0.4 or 0.9 % saline during individualized goal-directed fluid optimization. The primary outcome measure is a composite of death or major postoperative complications within 14 days following surgery.

The sample size will allow the detection of a 10 % absolute between-group difference in the primary outcome measure with a type 1 error rate of 5 % and power of 95 %, assuming a 5 % mortality rate and 20 % morbidity (thus 25 % for the composite endpoint).

**Discussion:**

The FLASH trial may provide important data on the efficacy and safety of commonly used fluid solutions and could have a significant impact on future treatment of surgical patients.

**Trial registration:**

ClinicalTrials.gov Identifier: NCT02502773. Registered 16 June 2015.

**Electronic supplementary material:**

The online version of this article (doi:10.1186/s13063-015-1085-3) contains supplementary material, which is available to authorized users.

## Background

Approximately 230 million patients undergo surgery worldwide each year, and recent data from the literature indicate that postoperative mortality ranges from 4 % after elective noncardiac surgery to 20 % in those undergoing emergency abdominal surgery [1, 2] and postoperative complications in 20 to 40 % of those patients considered to be at moderate-to-high risk of postoperative complications [3, 4].

Intravenous fluid therapy is one of the most common interventions in acute medicine [5] and a central aspect of perioperative management insofar as inappropriate fluid management in surgical patients is associated with increased postoperative morbidity, including postoperative organ dysfunctions [6–8], and mortality [9, 10]. Isotonic (0.9 %) saline is the most commonly used fluid worldwide [11]. Few studies, however, have examined the effects of particular types of fluids (crystalloid or colloid solutions) in surgical patients, the clinical practice in this specific area being mainly determined by clinician preference and local tradition [5, 12]; furthermore, much of the available data has been extrapolated from studies in critically ill patients. There is, however, increasing evidence that the type and dose of fluids administered independently affect patient outcomes [13].

Two large-scale pragmatic multicenter trials have recently been performed to compare crystalloids and different new-generation and low-molecular-weight preparations of hydroxyethyl starch (HES) in critically ill patients [14, 15]. The 6S study demonstrated a significant increase in 90-day mortality in patients with severe sepsis or septic shock treated with 6 % HES 130/0.4 rather than a balanced crystalloid [14]. In the CHEST study, no significant difference was found in the 90-day mortality, but the use of 6 % HES 130/0.42 was associated with an increased risk of renal replacement therapy compared with the use of 0.9 % saline [15]. The two studies showed no significant difference in short-term hemodynamic resuscitation endpoints. These data have led the Pharmacovigilance Risk Assessment Committee (PRAC) of the European Medicines Agency to suspend the authorization to use HES in cases of sepsis, burn injury or critically ill patients but have allowed the continued use of HES in surgical patients. Evidence is also emerging to suggest that administration of a large volume of 0.9 % saline may contribute to the development of hyperchloremic metabolic acidosis and AKI [16, 17]. The use of balanced rather than non-balanced crystalloid solutions has recently been proposed as a pragmatic alternative to 0.9 % saline, but only limited evidence is currently available concerning comparable efficacy and safety of use [5, 13] without demonstration of a clinical benefit in surgical patients [18, 19].

Several meta-analyses, including studies conducted on heterogeneous patient groups with a low risk of complications, have been published on colloid use in surgical patients and showed no difference in terms of mortality and postoperative acute kidney injury (AKI) between crystalloids and low molecular weight HES [20–22]. A growing body of evidence suggests that the timing of fluid administration is a central aspect of fluid therapy and that, in surgical patients, hemodynamic goal-directed therapy (GDT) to increase blood flow can reduce postoperative morbidity [4, 6–8] and mortality in the higher-risk groups of patients [9, 10, 23]. A central tenet of hemodynamic GDT is the individualization of fluid therapy by using explicit goals of care to allow early correction of fluid deficits and avoid excessive administration by fluid titration. The importance of individualizing fluid therapy has particularly been emphasized in a recent statement from the international Fluid Optimization Group and a workgroup from the 12th Acute Dialysis Quality Initiative (ADQI) [24, 25]. To date, however, few studies have examined the effect of the particular types of fluid used during hemodynamic therapy, and most studies have used colloid solutions; therefore, reliable conclusions cannot be drawn. Despite what may be inferred from physiological studies [26, 27], available data from HES use in ICU patients encourage the use of crystalloids as a first-line therapy for correction of hypovolemia during surgery. Crystalloids and colloids, however, may not be completely interchangeable. Greater fluid volumes have been suggested as necessary to achieve the same hemodynamic targets with crystalloids than with colloids, with reported crystalloid/colloid ratios ranging from 1.5 to 4 [28–30]. Administration of excessive fluid during surgery is associated with an increased risk of tissue edema, postoperative respiratory complications [31], renal dysfunction [32], longer hospital stay, and higher postoperative mortality [33]. An experimental study showed that, compared to a crystalloid-based goal-directed fluid therapy, a colloid-based goal-directed fluid therapy using HES 130/0.4 was associated with a reduction in the total volume of fluid infused and an improved microcirculatory blood flow and tissue oxygen pressure in the small intestine after abdominal surgery [34]. Two other randomized clinical trials also compared crystalloid with HES in patients at low risk of complications undergoing abdominal surgery. One concluded on better hemodynamic stability and reduced transfusion of blood products with HES [35], whereas the other found no difference in postoperative outcome despite a higher positive fluid balance in patients treated with crystalloids [36].

Taken together, two hypotheses can be put forward. A colloid-based goal-directed individualized fluid therapy using HES may improve outcome compared to 0.9 % saline or may promote postoperative organ dysfunction and reduced survival in moderate-to-high risk patients undergoing major surgery. The large number of patients for whom the question applies each year worldwide, current concerns about the risks related to the use of HES-based products in surgical patients, and the cost differences between HES and 0.9 % saline indicate the importance of evaluating the benefit/risk balance of these products and of evaluating the influence of the type of solution administered for the correction of hypovolemia during surgery on postoperative outcomes.

## Methods/Design

### Ethics

Written consent will be obtained from all participants. The Institutional Review Board of the University Hospital of Clermont-Ferrand (France) approved the trial. By 23 April 2015 the study had been approved for all centers by a central ethics committee (Comité de Protection des Personnes Sud-Est VI, Clermont-Ferrand, France) with the registration number IDRCB 2014-005575-84. The FLASH trial was registered on 16 July 2015 at http://www.clinicaltrials.gov with trial identification number NCT02502773.

### Trial design

The FLASH (fluid loading in abdominal surgery: saline versus hydroxyethyl starch) trial is an investigator-initiated, national, multicenter, parallel randomized controlled two-arm trial with concealed allocation of moderate-to-high risk patients 1:1 to individualized goal-directed fluid therapy during abdominal surgery using 6 % HES 130/0.4 in 0.9 % sodium chloride solution (Voluven, Fresenius Kabi, Bad Hamburg, Germany) or 0.9 % sodium chloride (saline).

### CONSORT diagram

**Fig. 1:**
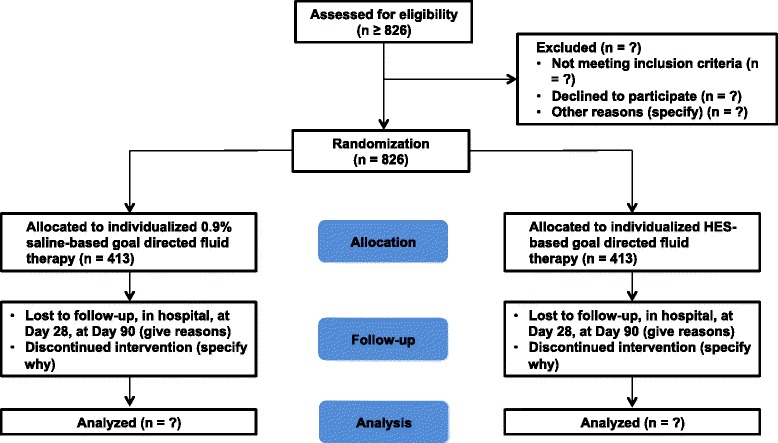
CONSORT flowchart illustrating the randomization and flow of patients in the study

The Consolidated Standards of Reporting Trials (CONSORT) diagram of FLASH is presented in Fig. 1.

### Selection of participants

Patients will be included in the FLASH trial if they comply with the indicated inclusion and exclusion criteria.

#### Inclusion criteria

For inclusion, adult patients must meet all the following criteria:

1. Elective or emergency abdominal surgery under general anesthesia
2. An anticipated operating time greater than or equal to 2 h
3. A moderate-to-high risk of postoperative complications defined by an AKI risk index ≥ class 3 (see Additional file 1) [37]
4. From whom written informed consent is obtainable either from the patient or from a legal representative (in case of non-elective surgery)

#### Exclusion criteria

Patients will be excluded for any of the following reasons:

1. Age <18 years
2. Patient with acute heart failure
3. Patient with acute coronary insufficiency
4. Patient with severe renal failure (defined by creatinine clearance <30 ml/min or requiring renal replacement therapy)
5. Patient with preoperative shock (defined by the need for vasoactive drugs before surgery)
6. History of allergy with the use of HES 130/0.4
7. Contraindication to the use of HES (as mentioned in the Summary of Product Characteristics for 6 % HES 130/0.4, Voluven, Bad Hamburg, Germany: sepsis, burnt patient, renal insufficiency or dialysis, cerebral hemorrhage, ICU patient, hypervolemia, lung edema, dehydration, severe hypernatremia or severe hyperchloremia, severe hepatic insufficiency, congestive heart failure, severe coagulopathy, or organ transplant)
8. Patient’s or relative’s refusal to participate
9. Parturient or breast-feeding women
10. Protected major (guardianship)

### Randomization and blinding

Randomization will be conducted over a dedicated, password-protected, SSL-encrypted website (EOL, Medsharing, Fontenay-sous-Bois, France) to allow immediate and concealed allocation. Each patient will be given a unique patient-number and a randomization number. The allocation sequence will be generated with the use of a minimization algorithm stratified according to center and timing of the surgical procedure (elective or emergency). The participant allocation will be carried out by local investigators who will log into the randomization system using a personal ID code and will enter any relevant information (including weight to calculate the maximum daily dose of trial fluid).

Trial fluids (HES and 0.9 % saline) are visually identical and consist in 500-ml Freeflex fluid bags containing HES or 0.9 % saline. Trial fluids will be blinded and identified only by a unique number from the specific trial site. The initial and any subsequent allocation of trial fluids will be determined by a web-based randomization system. The logistics of the trial fluid distribution to each of the 20 participating centers that are anticipated to be recruiting will be coordinated by the pharmacy of the coordinating center. The receipt, storage and dispensing of the blinded trial fluids will be conducted by the pharmacy department in each individual trial site. Each trial site will have a sufficient number of bags of trial fluid to be allocated to the included patients. The initial and any subsequent allocation of trial fluids will be determined by the web-based randomization system at each site. This will ensure that the patient only receives the trial fluid that he/she was randomized to receive. The information regarding which codes correspond to what treatment will be maintained in a secure location at the coordinating center. All staff at the participating trial sites and the coordinating center will be blinded to the treatment allocation.

### Trial interventions

All included patients will be allocated to one of the following two study groups:

1. Crystalloid-based GDT. Patients in this group will be receive individualized goal-directed fluid administration of 0.9 % saline (Sodium chloride, 500 mL FreeFlex™, Fresenius Kabi, Bad Hamburg, Germany) to optimize stroke volume.
2. Colloid-based GDT. Patients in this group will receive individualized goal-directed fluid administration of 6 % HES 130/0.4 (Voluven™, Fresenius Kabi, Bad Hamburg, Germany) to optimize stroke volume.

**Fig. 2:**
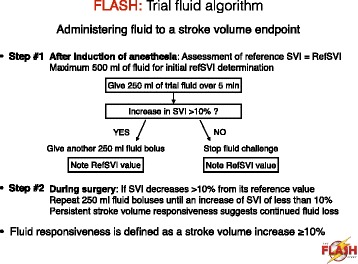
Trial fluid administration according to the stroke volume-guided hemodynamic therapy algorithm. *Abbreviations*: *refSVI* reference value of SVI, *SVI* stroke volume index

Trial fluid is to be used for volume expansion during surgery and for a maximum of 24 h after surgery. In each group, patients will receive 250 ml fluid challenges of the trial fluids, within a period of 5 min, aiming to maximize stroke volume as suggested by French and international recommendations for perioperative hemodynamic optimization [24, 38]. In order to ensure a standardized approach to fluid administration, no more than 500 ml of trial fluid will be administered for initial determination of the maximal value of the stroke volume. Once the maximal value of the stroke volume has been determined after induction of anesthesia, stroke volume will be maintained throughout the intervention period with subsequent boluses of trial fluids as required (Fig. 2). The clinical decision to administer trial fluids during the first 24 h after surgery, as with other aspects of postoperative care (see below), will be determined by the treating clinician according to routine clinical practice and the expertise of the staff at each center. In each group, the maximum dose of trial fluid will be 30 ml/kg/day to comply with the maximum registered dose of 6 % HES 130/0.4. If the upper limit of the trial fluid is exceeded, unmasked 0.9 % saline will be used in all patients.

### Standard procedures

In all patients, lactated Ringer’s solution will be administered at a maximum infusion rate of 4 ml/kg/h to satisfy the maintenance fluid requirements during surgery.

Decisions about all other aspects of patient care during the intraoperative and postoperative periods (especially general anesthesia, postoperative pain management and physiotherapeutic procedures) will be performed according to the expertise of the staff at each center and to routine clinical practice to minimize interference with the trial intervention. Nevertheless, trial investigators will be strongly encouraged to apply standard measures to avoid extremes of clinical practice, as follows:

1. Mean arterial pressure will be maintained at 60 mmHg or higher.
2. Oxygenation (pulse oximetry) will be maintained at 94 % or higher.
3. Blood products will be given to maintain hemoglobin at level greater than 8 g/dl (in patients with no history of ischemic heart disease or ≥10 g/dl otherwise).
4. Normothermia and normoglycemia will be maintained throughout the surgical period.
5. Lung-protective ventilation will be used during surgery.
6. Enhanced recovery after surgery (ERAS) guidelines will be applied.
7. Appropriate prophylactics antibiotics will be used as recommended.

### Outcomes

The primary outcome measure will be a composite of mortality or major postoperative complications occurring by day 14 after surgery, defined as one or more of the following:

1. Acute kidney injury (defined by KDIGO stage 1 or higher)
2. Pulmonary complication (defined by the need for noninvasive or invasive ventilatory assistance for postoperative acute respiratory failure)
3. Cardiovascular complication (defined by the development of acute heart failure)
4. Infectious complication (defined by the development of sepsis, severe sepsis or septic shock)
5. Surgical complication (defined as the need for surgical reoperation)

In addition, each component of the primary outcome measure will be analyzed separately.

Secondary outcome measures will be as follows:

1. Postoperative complications evaluated separately within 14 days:
  - Kidney dysfunction: oliguria (24-h urine output < 500 ml), KDIGO score
    (KDIGO categories [39]:
    KDIGO stage 1: increase in serum creatinine × 1.5 to 1.9 OR ≥26.5 μmol/l from baseline OR urine output <0.5 ml/kg/h for 6 to 12 h
    KDIGO stage 2: increase in serum creatinine × 2.0 to 2.9 from baseline OR urine output <0.5 ml/kg/h for ≥12 h
    KDIGO stage 3: increase in serum creatinine × 3.0 from baseline OR ≥353 μmol/l OR initiation of renal replacement therapy OR urine output <0.3 ml/kg/h for ≥24 h OR anuria ≥12 h)
  - Cardiovascular complications: cardiac arrhythmia, acute heart failure, myocardial infarction, or pulmonary embolism
  - Pulmonary complications: postoperative hypoxemia, postoperative pneumonia, need for intubation and invasive mechanical ventilation or postoperative noninvasive ventilation, postoperative ARDS, and days alive without ventilation (ventilator-free days)
  - Postoperative systemic inflammatory response syndrome (SIRS) score [40]
  - Infectious complications: surgical site infection, intra-abdominal abscess, postoperative peritonitis, sepsis, severe sepsis or septic shock
  - Surgical complications: anastomotic leak, reoperation or endoscopic drainage
  - Sepsis-related organ failure assessment (SOFA, modified from [41], see Additional file 2) score (excluded Glasgow Coma Score)
  - Total fluid volume (0.9 % saline and HES 130/0.4) administered during the surgical period and the first 24 postoperative hours
  - Volume of blood loss and number of units of packed red blood cells administered during the operative period and the first 24 postoperative hours
  - Time to return of bowel function (flatus and stool)
  - Unexpected ICU admission (or readmission) within 28 days following surgery
  - Duration of hospital stay: high dependency unit (HDU), intensive care unit (ICU), hospital stay
  - All-cause mortality at Day 28 and 3 months

### Patient withdrawal

Trial fluid is to be used only during the surgical period and for a maximum of 24 h after surgery. Nevertheless, a participant or a patient’s relative who no longer agrees to participate in the clinical trial can withdraw the informed consent at any time without need of further explanation. Patients who are withdrawn from the trial fluid-treatment protocol will be followed up and analyzed as with the remaining patients. In order to conduct intention-to-treat analyses with as little missing data as possible, it is in the interest of the trial to collect as much data from each participant as possible. Therefore, the investigator may ask the participant and/or relatives which aspects of the trial he/she wishes to withdraw from (participation in the remaining follow-up assessments or use of already collected data) and, whenever possible, and the participant will be asked for permission to obtain data for the primary outcome measure. If this person declines, no more data will be collected, and new patients will be randomized to obtain the full sample size. All randomized patients will be reported, and all data available with consent will be used in the analyses. If appropriate, missing data will be handled in accordance with multiple imputation procedures if missing data are greater than 5 %.

### Safety

All adverse events thought to be related to the trial fluids will be reported to the trial coordinating center. According to the French Public Health Code, all suspected unexpected serious adverse events will be reported to the ANSM. In addition, this information will be submitted to the data monitoring and safety committee (DMSC). The DMSC is independent of the trial investigators and will perform an ongoing review of safety parameters and overall study conduct. The DMSC is comprised of two independent experts in large-scale clinical trials and one independent statistician.

The DMSC will be responsible for safeguarding the interests of trial participants, assessing the safety and efficacy of the interventions during the trial, and for monitoring the overall conduct of the clinical trial. To contribute to enhancing the integrity of the trial, the DMSC may also formulate recommendations relating to the recruitment/retention of participants, their management, improving adherence to protocol-specified regimens and retention of participants, and the procedures for data management and quality control. The DMSC will provide recommendations about stopping or continuing the trial to the Steering Committee (SC) of the FLASH trial. Stopping rules will be based on the following:

1. Group-difference in all causes mortality or
2. Group-difference in postoperative AKI (requirement for renal replacement therapy)

The DMSC will be advisory to the SC. The SC will be responsible for promptly reviewing the DMSC recommendations, to decide whether to continue or terminate the trial, and to determine whether amendments to the protocol or changes in trial conduct are required.

### Statistics

Analysis will be by intention-to-treat (ITT) on data from the modified-ITT population (see Additional file 3). Unadjusted chi-square test or Fisher’s exact test will be used for the primary outcome analysis. Adjusted analysis will be conducted with the use of robust random effects Poisson generalized-linear regression (1) to take into account adjustment on possible confounding covariates selected according to clinical relevance and stratification variables and (2) to consider within and between center variability. Data will be presented as relative risks and 95 % confidence intervals.

Regardless of the results from the analysis of the composite outcome, each component of the composite primary outcome measure will be also analyzed separately using similar methods as described for the primary analysis. The Hochberg procedure will be used to adjust for multiple testing of components of the composite primary outcome. A chi-square test (or Fisher’s exact test, as appropriate) will be used for secondary binary outcomes. Continuous variables will be compared with the use of the unpaired *t*-test or Mann–Whitney *U* test as appropriate. The Shapiro-Wilk test will be used to assess homoscedasticity. Adjusted analyses will be performed using the same adjustments variables. Time-to-event curves will be calculated with the use of the Kaplan-Meier method. Longitudinal analysis using mixed models will be used to take into account within and between subject variability.

Per protocol analyses will be performed on the primary outcome and the most important secondary outcomes. Missing data will be handled in accordance with the multiple imputation method (STATA command mi) if missing data are greater than 5 % [42].

A total of 2 × 413 patients will be needed to have 95 % power to show an absolute between-group difference of 10 % in the primary outcome measure at a two-sided alpha level of 0.05, assuming a 5 % mortality rate and 20 % morbidity at 14 days after surgery (thus, 25 % for the composite endpoint). Data from the literature have shown that postoperative mortality ranges from 4 % in patients undergoing elective noncardiac surgery to 20 % in those undergoing emergency abdominal surgery [1, 2, 43], with an average rate of postoperative complications following major abdominal surgery of 20 to 40 % [3, 4].

Given current concerns related to possible adverse effects of IV therapy solutions (6 % HES and 0.9 % saline), an interim safety analysis will be performed after data for 210 and 420 patients have been obtained using the Lan and DeMets method (East software, Cytel Inc., Cambridge, MA, USA). The DMSC will recommend that the trial be stopped if it is found that the conduct of the trial compromises patient safety (a between-group difference in mortality or postoperative AKI).

All analyses will be conducted with Stata statistical software, version 12 (StataCorp. 2011. *Stata Statistical Software*: *Release 12*. College Station, TX: StataCorp LP). A two-sided P value of less than 0.05 will be considered for statistical significance.

### Data registration

Data will be entered into the web-based electronic case report form (eCRF) (EOL, Medsharing, Fontenay-sous-Bois, France) by trial or clinical personnel under the supervision of the trial site investigators at each participating center. Data collection will be monitored by trained research coordinators.

The following data will be registered:

Pre-randomization and baseline characteristics: Demographic data (age, height, weight, gender, and body mass index); co-morbidities (hypertension Y/N, renal dysfunction Y/N, chronic heart failure Y/N, diabetes mellitus Y/N, malnutrition Y/N, chronic alcoholism Y/N, and active smoking Y/N); American Society of Anesthesiologists (ASA) physical status; surgical operation for malignancy Y/N; preoperative use of nephrotoxic drugs (NSAIDs) Y/N; preoperative use of angiotensin-converting enzyme inhibitors and/or angiotensin II subtype receptor antagonists Y/N; preoperative administration (type and volume) of intravenous fluids Y/N; preoperative administration (and volume) of blood products Y/N; and routine biological data, including baseline serum creatinine.

At randomization: AKI risk index (class), urgency of surgery (elective or non-elective surgery) (stratification variable).

During the surgical procedure, the following data will be collected:

1. Anesthetic data: type and doses of hypnotics, opioids and muscle relaxants; duration of anesthesia; total volume of maintenance fluid; total volume of trial fluids (aliquots of 250 ml); use (Y/N) and total volume of 0.9 % saline (if volume of trial fluid greater than 30 ml/kg); total number blood products; ventilator settings (tidal volume, PEEP, FiO_2_); baseline (and then hourly) values for bispectral index or entropy; blood pressures (systolic, diastolic and mean) and stroke volume index (SVI); type of hemodynamic monitor (esophageal Doppler Y/N, pulse contour Y/N, other); total number of episodes of hypovolemia; need for administration (Y/N) and infusion rate of vasoactive drugs (ephedrine hydrochloride, noradrenaline, other); and hourly urine output.
2. Surgical data: type of surgery, duration of surgery, surgical technique (laparoscopy Y/N, laparotomy Y/N), total volume of blood losses, and surgical complications Y/N.

From randomization to postoperative Day 1 (07.59 AM): total volume of trial fluids (aliquots of 250 ml); use (Y/N) and total volume of 0.9 % saline (if volume of trial fluid greater than 30 ml/kg); total number blood products and blood losses; need for mechanical invasive- or non-invasive ventilation; hourly values of blood pressures and urine output; need for administration (Y/N); and infusion rate of vasoactive drugs.

Daily from postoperative Day 1 (08.00 AM) to Day 7 after surgery (or hospital discharge):

1. Postoperative care pathway (surgical ward Y/N, HDU Y/N, and ICU Y/N)
2. Epidural analgesia Y/N
3. Daily volume of trial fluid (only on postoperative Day 1) and maintenance fluids including oral and parenteral nutrition, urine output and calculated fluid balance
4. Daily lowest values for heart rate, blood pressure, peripheral O2 saturation, respiratory rate, temperature
5. Blood glucose level
6. Results of samples of plasma creatinine and creatinine clearance, plasma lactate, CRP, bilirubin (standard laboratory values)
7. SIRS score
8. KDIGO score
9. Variables for SOFA scoring not covered above (until Day 5)
10. Postoperative complications (Y/N, type, and date of diagnosis)
11. Unexpected ICU admission Y/N
12. Length of stay in HDU, ICU, and surgical ward
13. Date of hospital discharge
14. Death (Y/N and date)

Fourteen days after surgery:

1. Postoperative complications (Y/N, type, and date of diagnosis)
2. Need for renal replacement therapy on that day Y/N, and total days of RRT (between first and last treatment session)
3. Unexpected ICU admission (Y/N, date of admission)
4. Length of stay in HDU, ICU, surgical ward
5. Date of hospital discharge (as obtained from hospital notes)
6. Survival status (If the patient is deceased, date of death)

If the patient is still present on Day 14, follow up will continue until hospital discharge.

Twenty-eight days after surgery:

1. Postoperative complications (Y/N, type, and date of diagnosis) from Day 14 to Day 28 after surgery
2. Survival status

Ninety days after surgery:

1. Survival status (if the patient is deceased, date of death)

### Data handling and retention

Data will be handled according to French law. All original records (including consent forms, reports of suspected unexpected serious adverse events, and relevant correspondences) will be archived at the trial sites for 15 years. The clean trial database file will be anonymized and maintained for 15 years.

### Enrollment and timeline

The patients are expected to be included from 20 French university and non-university hospitals during a 2-year period starting in September 2015.

Each participating center has to include two patients per month (holidays excluded) to finish inclusion in 2 years.

2013–2015: Protocol, approvals from ethics committee, and trial tool development (eCRF, randomization system)

2015 to 2017: Inclusion of patients

2017: Cleaning and closure of the database.

Early 2018: Data analyses and writing of the manuscript, and submission for publication.

### Publication plan

The trial is registered on http://www.clinicaltrials.gov. Upon trial completion, the main manuscript will be submitted to one of the major clinical journals regardless of the results. All trial sites, including patients, will be acknowledged, and all investigators at these sites will appear with their names under ‘the FLASH investigators’ in an Appendix to the final manuscript. The FLASH trial Steering Committee will grant authorship in adherence to the Vancouver guidelines and number of patients enrolled by the individual investigator. If a trial site investigator is to gain authorship, the site has to include 50 patients or more. If the site includes 80 patients or more, two authorships will be granted. A writing committee will be composed of members of the steering committee and investigators to define the order of authors of any publications.

The listing of authors will be as follows: E Futier (principal investigator) will be responsible for the writing of the manuscript and will be the first author, then other members of the Steering Committee and trial site investigators depending on the number of included patients per site (trial site investigator with the greater number of inclusion will be second author), B Pereira (biostatistician) will be second to last author, S Jaber will appear as the last author and then ‘for the FLASH trial investigators’ will be added.

### Finances

The FLASH trial is funded by the Agence Nationale de Sécurité du Médicament (ANSM), the Société Française d’Anesthesie Réanimation (SFAR) and Clermont-Ferrand University Hospital.

Fresenius Kabi provides the blinded study treatment for the conduct of the trial and will manage the warehousing and distribution of the study treatment from the manufacturing plant in France to the coordinating center (Clermont-Ferrand University Hospital). All blinded study treatment will be manufactured according to Good Manufacturing Practice requirements.

Management and logistic of the trial fluid distribution to each of the 20 participating centers that are anticipated to be recruiting will be coordinated using the web-based randomization system by the pharmacy of the Clermont-Ferrand University Hospital. The receipt, storage and dispensing of the blinded trial fluids will be the responsibility of the pharmacy department in each individual trial site. This will be performed in accordance with accredited standards for routine pharmacy practice.

Funding sources have no influence on trial design, trial conduct, data handling, data analysis or writing of the manuscript.

### Perspectives

Million of patients undergo major abdominal surgery worldwide each year. Inappropriate (excessive or insufficient) perioperative fluid therapy may be associated with significant postoperative complications that adversely affect both short-term and long-term survival and increase the costs of healthcare. Few studies, however, have examined the effects of particular types of fluids (crystalloid or colloid solutions) in surgical patients, and much of the available data are extrapolated from studies in critically ill patients. As far as the investigators are aware, no other large RCTs are assessing the efficacy and safety of 0.9 % saline or 6 % HES with the goal of hemodynamic optimization during major surgery.

## Discussion

Performing the FLASH trial is in line with conclusions from the 2013 recommendations of the European Medicines Agency’s Pharmacovigilance Risk Assessment Committee on the lack of robust safety data of HES in patients undergoing surgical procedures and the need to perform additional studies with HES solutions in surgical patients, especially in those at high risk of postoperative complications.

HES solutions are widely used in surgical patients as a component of hemodynamic therapy for fluid optimization. Isotonic saline, which is the most widely used fluid worldwide, is a commonly accepted and effective alternative but may be associated with potential nephrotoxicity. Whatever the results of the FLASH trial, this information will provide urgently needed data on efficacy and safety of commonly used fluid solutions, which could have a significant impact on the future treatment of surgical patients.

### Trial status

The FLASH trial is not yet recruiting.

## Additional files

Additional file 1:
**Acute kidney injury (AKI) risk index.** (PDF 50 kb) Additional file 2:
**Sepsis-related Organ Failure Assessment (SOFA) score.** (PDF 68 kb) Additional file 3:
**FLASH trial statistical analysis plan.** (PDF 78 kb)

## Abbreviations

AKI: acute kidney injury
ANSM: Agence National de Sécurité du Médicament
ARDS: acute respiratory distress syndrome
eCRF: electronic (web-based) case report form
ERAS: enhanced recovery after surgery
DMSC: data monitoring safety committee
GDT: goal-directed therapy
HES: hydroxyethyl starch
HDU: high dependency unit
ICU: intensive care unit
KDIGO: kidney disease improving global outcomes
RCT: randomized controlled trial
SC: steering committee
SIRS: systemic inflammatory response syndrome
SOFA: Sepsis-related organ failure assessment
