# Supplementary material for: Fluid loading in abdominal surgery - saline versus hydroxyethyl starch (FLASH Trial): study protocol for a randomized controlled trial
Source: Trials. 2015 Dec 21;16:582. doi: 10.1186/s13063-015-1085-3 (PMC4687283; doi:10.1186/s13063-015-1085-3)
Supplement: Additional file 1: — Acute kidney injury (AKI) risk index. (PDF 50 kb) [file 13063_2015_1085_MOESM1_ESM.pdf]

Additional file 1: Acute Kidney Injury (AKI) risk index [37]

Individual preoperative risk factors included in the general surgery acute kidney injury risk index:

- Age ≥ 56 years
- Male sex
- Active Congestive Heart Failure
- Ascites
- Hypertension
- Emergency Surgery
- Intraperitoneal Surgery
- Renal insufficiency – mild or moderate
  (Serum Creatinine >105 µmol/L or 1.2 mg/dL)
- Diabetes Mellitus – Oral or insulin therapy

Five general surgery acute kidney risk index classes are based on the number of risk factor the patient possesses:

Risk class				
**Class I**	**Class II**	**Class III**	**Class IV**	**Class V**
0 – 2 risk factors	3 risk factors	4 risk factors	5 risk factors	+ than 6 risk factors

Only patients with a risk class of 3 or more will eligible for inclusion in the FLASH trial
