# Supplementary material for: Fluid loading in abdominal surgery - saline versus hydroxyethyl starch (FLASH Trial): study protocol for a randomized controlled trial
Source: Trials. 2015 Dec 21;16:582. doi: 10.1186/s13063-015-1085-3 (PMC4687283; doi:10.1186/s13063-015-1085-3)
Supplement: Additional file 2: — Sepsis-related Organ Failure Assessment (SOFA) score. (PDF 68 kb) [file 13063_2015_1085_MOESM2_ESM.pdf]

Additional file 2: SOFA scoring (excluding Glasgow Coma Score) - the most deranged value recorded in the previous 24 h will used [42]

ORGAN SYSTEM	0	1	2	3	4	Organ scores
**Respiration**<br>PaO_2 / FiO_2 (in mmHg)	>400	301 – 400	<301 (without respiratory support*)	101 – 200 (with respiratory support*)	≤100 (with respiratory support*)	
**Coagulation**<br>Platelets (x 10^3/mm^3)	>150	101 – 150	51 – 100	21 – 50	≤20	
**Liver**<br>Bilirubin (μmol/l)	<20	20 – 32	33 – 101	102 – 204	>204	
**Cardiovascular**<br>Hypotension	Mean arterial pressure (MAP) > 70 mmHg	MAP <70 mmHg	dopamine ≤ 5.0 (dose in μg/kg/min)	dopamine >5.0 (dose in μg/kg/min)	dopamine >15.0 (dose in μg/kg/min)	
			or any dose dobutamine	or noradrenalin ≤0.1<br>or adrenalin ≤0.1	or adrenalin >0.1<br>or noradrenalin >0.1	
**Renal**<br>Creatinine (μmol/l)	<110	110 – 170	171 – 299	300 – 440	> 440	
**OR** Urine output				or <500 ml / day	or <200 ml / day	

If a value has not been measured, the score 0 should be given. *Respiratory support is defined as any form of invasive or non-invasive ventilation including continuous positive airway pressure delivered through mask or tracheotomy
