# Supplementary material for: Fluid loading in abdominal surgery - saline versus hydroxyethyl starch (FLASH Trial): study protocol for a randomized controlled trial
Source: Trials. 2015 Dec 21;16:582. doi: 10.1186/s13063-015-1085-3 (PMC4687283; doi:10.1186/s13063-015-1085-3)
Supplement: Additional file 3: — FLASH trial statistical analysis plan. (PDF 78 kb) [file 13063_2015_1085_MOESM3_ESM.pdf]

Additional file 3

FLASH-trial Statistical Analysis Plan

Populations

Intention-to treat population: All randomized patients
This population will not be analyzed in the FLASH trial

Modified intention-to-treat population: All randomized patients except patients who

- Withdrew consent for the use of data

OR

- Would never had any of the intervention (masked fluid trial)

Per-protocol population: All randomized patients except patients having one or more major protocol violations defined as

- Patients who would not be eligible for randomization according to inclusion/non-inclusion criteria

OR

- Patients who would never had the intervention (masked trial fluid)

OR

- Patients who accidentally would have received the wrong intervention

OR

- Patients in whom surgical intervention would not have been done (for example, surgical removal would have been impossible because of extensive tumor progression)

OR

- Patients who would have be withdrawn from the protocol

Analyses

Analyzes will be conducted, first, on data from the modified intention-to-treat (ITT) population and, second, in the per-protocol population.

Primary analysis:
Unadjusted Chi-square test (or Fisher's exact test as appropriate) for binary outcome. For rate data the generalized linear (STATA command glm) model will be used with Poisson distribution, link=log and offset.
Results will be expressed as Relative Risks and 95% confidence intervals.

Secondary analysis:
Multiple (logistic) regression for the primary outcome with the following covariates (criterion for entering variables tested in the model will be selected if $P<0.10$ and according to clinically relevant covariates with anticipated relationship with outcome):

- Binary covariates
    - Trial center (stratification variable)
    - Gender M/F
    - Preoperative renal dysfunction at randomization (baseline serum creatinine >105 µmol/l) Y/N
    - Congestive Heart Failure Y/N
    - Hypertension Y/N
    - Diabetes mellitus Y/N
    - Cancer Y/N
    - Surgical technique (laparotomy Y/N)

- Continuous covariate
    - Age
    - Urgency of surgery (elective vs non-elective) (stratification variable)
    - Surgical procedure category
    - Surgical duration
    - Blood transfusion – Number of unit
    - Hypovolemia – Number of episodes
    - Planned ICU/HDU admission following surgery
- Ordinal covariates
    - ASA score
    - AKI risk index class

Each component of the composite primary outcome measure will be analyzed separately using similar methods as described for the primary analysis. A chi-square test (or Fisher's test, as appropriate) will be used for secondary binary outcomes. The Hochberg procedure will be used to adjust for multiple testing of components of the composite primary outcome.

Continuous variables will be presented as mean and standard deviations (as median and quartiles, otherwise) and will be compared with the use of the unpaired *t* test or the Mann-Whitney *U* test when appropriate. The Shapiro-Wilk test will be used will be used to assess normality, and the Fisher-Snedecor test to assess homoscedasticity. Adjusted analyses will be conducted using the same adjustment variables.

Time-to-event curves will be calculated with the use of the Kaplan-Meier method.

Longitudinal analysis using mixed models will be used to take into account between and within subject variability.

Level of statistical significance for all analyses: P = 0.05

Outcomes

Primary outcome measure:
The composite outcome measure of mortality or major postoperative complications by day 14 after surgery. Major postoperative complications include one or more of the following: acute kidney injury (defined as KDIGO stage 1 or higher), need for non-invasive or invasive ventilator assistance for postoperative respiratory failure, acute heart failure, severe infectious complication (defined as postoperative sepsis, severe sepsis or septic shock) and need for surgical reoperation.

Secondary outcome measures:

- Kidney dysfunction: oliguria (24-hour urine output < 500 ml), KDIGO score [39], need for renal replacement therapy within 14 days after surgery
- Cardiovascular complications: cardiac arrhythmia, acute heart failure, myocardial infarction, pulmonary embolism within 14 days after surgery
- Pulmonary complications: postoperative hypoxia, postoperative pneumonia, need for tracheal intubation and invasive mechanical ventilation or noninvasive ventilation, postoperative ARDS, days alive without ventilation within 14 days after surgery
- Infectious complications: surgical site infection, intra-abdominal abscess, postoperative peritonitis, sepsis, severe sepsis and septic shock within 14 days after surgery
- Surgical complications: anastomotic leak, need for reoperation or endoscopic drainage within 14 days after surgery
- Sepsis-related organ failure assessment (SOFA) score [42] (without cerebral component, see Additional file 2) within day 7 after surgery
- Postoperative systemic inflammatory response syndrome (SIRS) score [40], within day 7 after surgery
- Total volume of fluid (0.9% saline and HES 130/0.4) infused during the surgical period and the first 24 postoperative hours
- Volume of blood loss and number of units of packed red blood cells administered during the operative period and the first 24 postoperative hours
- Time to return of bowel function (flatus and stool)
- Unexpected ICU admission (or readmission) within 28 days following surgery
- Duration of hospital stay: high dependency unit (HDU), intensive care unit (ICU), hospital stay until hospital discharge or censoring
- All-cause mortality at Day-28 and 3 months
- Time to death or censoring

Missing data

If missing data are greater than 5%, an additional analysis will be performed using the multiple imputation method (STAT command mi).
